# Beyond Sagittal and Vertical: Transverse Constriction as a Feature of the Collapsed Arch in Angle Class II Post-Extraction Retreatment Patients: A Retrospective CBCT Pilot Study

**DOI:** 10.3390/healthcare14152350

**Published:** 2026-08-02

**Authors:** Yinti Pan, Changtao Qin, Anjie Guo, Xin Sun, Xingyu Lu, Yanling Xie, Yue Feng, Zhixing Chen, Shuixue Mo

**Affiliations:** 1 College of Stomatology, Guangxi Medical University, Nanning 530021, China; panyinti@sr.gxmu.edu.cn (Y.P.); 202400267@sr.gxmu.edu.cn (C.Q.); anjieguo@sr.gxmu.edu.cn (A.G.); sunx0723@outlook.com (X.S.); luxy@sr.gxmu.edu.cn (X.L.); xyl113226339@163.com (Y.X.); fengyue_gxmu@163.com (Y.F.); 2 Guangxi Clinical Research Center for Craniofacial Deformity, Nanning 530021, China; 3 Guangxi Key Laboratory of Oral and Maxillofacial Rehabilitation and Reconstruction, Nanning 530021, China

**Keywords:** arch width, CBCT, dental arch form, orthodontic retreatment, premolar extraction

## Abstract

**Highlights:**

**What are the main findings?**

- This preliminary study quantitatively describes a distinct “arch collapse” morphology in a cohort of Angle Class II patients with a history of four-premolar extractions. This pattern is characterized by severe transverse posterior constriction (4.17–5.37 mm narrower), an elongated and tapered anterior arch, and abnormal tooth inclination.
- The initial findings suggest that comprehensive retreatment using a passive self-ligating bracket system may improve most arch dimensions and occlusal parameters to levels comparable to first-time treatment patients; however, the mandibular interpremolar width remained significantly narrower.

**What are the implications of the main findings?**

- The “arch collapse” phenotype observed in post-extraction retreatment patients is likely a biomechanical consequence of anchorage loss during initial treatment failure. Combining self-ligating bracket alignment with selective maxillary TAD-assisted distalization presents a potentially viable corrective strategy for this specific population.
- The incomplete correction of the mandibular interpremolar width reflects inherent anatomical constraints in the posterior mandible, highlighting the necessity for specific biomechanical interventions and strict retention protocols, which warrants further validation in large-scale prospective cohorts.

**Abstract:**

**Background**: Orthodontic retreatment patients with a history of premolar extraction for Angle Class II malocclusion exhibit distinct dental arch morphologies; however, studies characterizing these features remain limited. This pilot study aimed to objectively assess baseline dental arch morphology and preliminary retreatment-related changes in this specific patient population. **Methods**: In this retrospective pilot investigation, a total of 21 subjects were consecutively enrolled and divided into two groups according to orthodontic history: the Retreatment Group (*n* = 9, patients with Class II malocclusion and a history of prior orthodontics with four-premolar extractions) and the Control Group (*n* = 12, first-time orthodontic patients with Class II malocclusion undergoing identical four-premolar extraction therapy during the current treatment). All participants were treated with a standardized 0.022-inch MBT self-ligating system. Pre-treatment (*T*0) and post-treatment (*T*1) CBCT data were analyzed to evaluate the following variables: intercanine width, interpremolar width (IPMW), intermolar width (IMW), anterior arch depth, total arch depth, arch perimeter, intercanine angle, mesiodistal angulation (MA), buccolingual inclination (BI), depth of the curve of Spee, overbite, and overjet. Arch width was designated as the primary outcome. Student’s *t*-tests were used for statistical analysis (α = 0.05). **Results**: At *T*0, the Retreatment Group had narrower posterior arches than the controls (*p* < 0.01; *Hedges’ g* = 1.55–1.94), with IPMW smaller by 4.9 ± 1.11 mm (maxillary) and 5.37 ± 1.25 mm (mandibular), and IMW smaller by 4.85 ± 1.06 mm (maxillary) and 4.17 ± 1.13 mm (mandibular). They also had greater overbite and overjet, greater maxillary anterior depth, smaller intercanine angle, and lower incisor BI and canine MA (*p* < 0.05). From *T*0 to *T*1, the main treatment changes included arch width expansion and adjustments in MA and BI. At *T*1, most parameters were similar between groups, but mandibular IPMW remained narrower in the Retreatment Group (*p* < 0.01). **Conclusions**: The Retreatment Group showed a distinct “arch collapse” pattern characterized by posterior constriction, increased overbite and overjet, an elongated and tapered arch, and abnormal incisor torque and canine angulation. Orthodontic retreatment corrected most of these features, but mandibular IPMW correction remained limited.

## 1. Introduction

Against the backdrop of a growing global population of orthodontic patients, adult orthodontic retreatment is emerging as a distinct clinical challenge [1]. The substantial demand for orthodontic retreatment is supported by studies indicating that 60.10% of previously treated Chinese youth and 43.67% of patients in Saudi Arabia require secondary intervention [2,3]. Compared to first-time orthodontic patients, those seeking retreatment typically present with more complex psychological expectations and greater anatomical constraints [4].

The ideal dental arch form serves as the foundation for occlusal function and long-term stability [5,6,7,8]. In the literature, malocclusion observed in orthodontic retreatment patients is defined as “secondary malocclusion,” a morphological pattern that differs from primary malocclusion in both etiology and biomechanics [1,9,10], particularly in Angle Class II malocclusion with premolar extractions. Many studies have reported a “roller coaster effect” in these patients, characterized by structural decompensation such as distal tipping of anterior crowns, mesial tipping of posterior teeth, and concomitant deep overbite and overjet [11,12,13,14].

However, current research on structural decompensation focuses predominantly on the sagittal and vertical dimensions [11,12], with a notable lack of quantitative data on transverse arch morphology. Furthermore, existing evidence on orthodontic retreatment is largely limited to individual case reports or investigations of anchorage loss management within primary treatment cohorts. Systematic evaluations of dental arch morphology in patients specifically seeking secondary Class II correction remain sparse. Traditional methods such as model-based or two-dimensional radiographic analyses used in previous studies can assess only crown position and fail to reveal the true spatial orientation of tooth roots within the three-dimensional alveolar bone environment [15,16]. In contrast, cone-beam computed tomography (CBCT) enables precise evaluation of transverse arch dimensions and dental axial inclination, providing a reliable and reproducible basis for investigating three-dimensional arch morphology [17,18].

Therefore, this retrospective cohort study utilizes CBCT technology to systematically evaluate the dental arch morphology of Angle Class II retreatment patients with a history of four-premolar extraction, compared to first-time orthodontic patients. Using a standardized three-dimensional measurement method, this study takes dental arch width as the primary outcome to analyze transverse dimensional differences between groups. Dental arch depth and perimeter, intercanine angle, tooth inclination (mesiodistal angulation and buccolingual inclination), and occlusal parameters (overjet and overbite) are comprehensively evaluated as secondary outcomes.

## 2. Materials and Methods

### 2.1. Study Design

**Figure 1:**
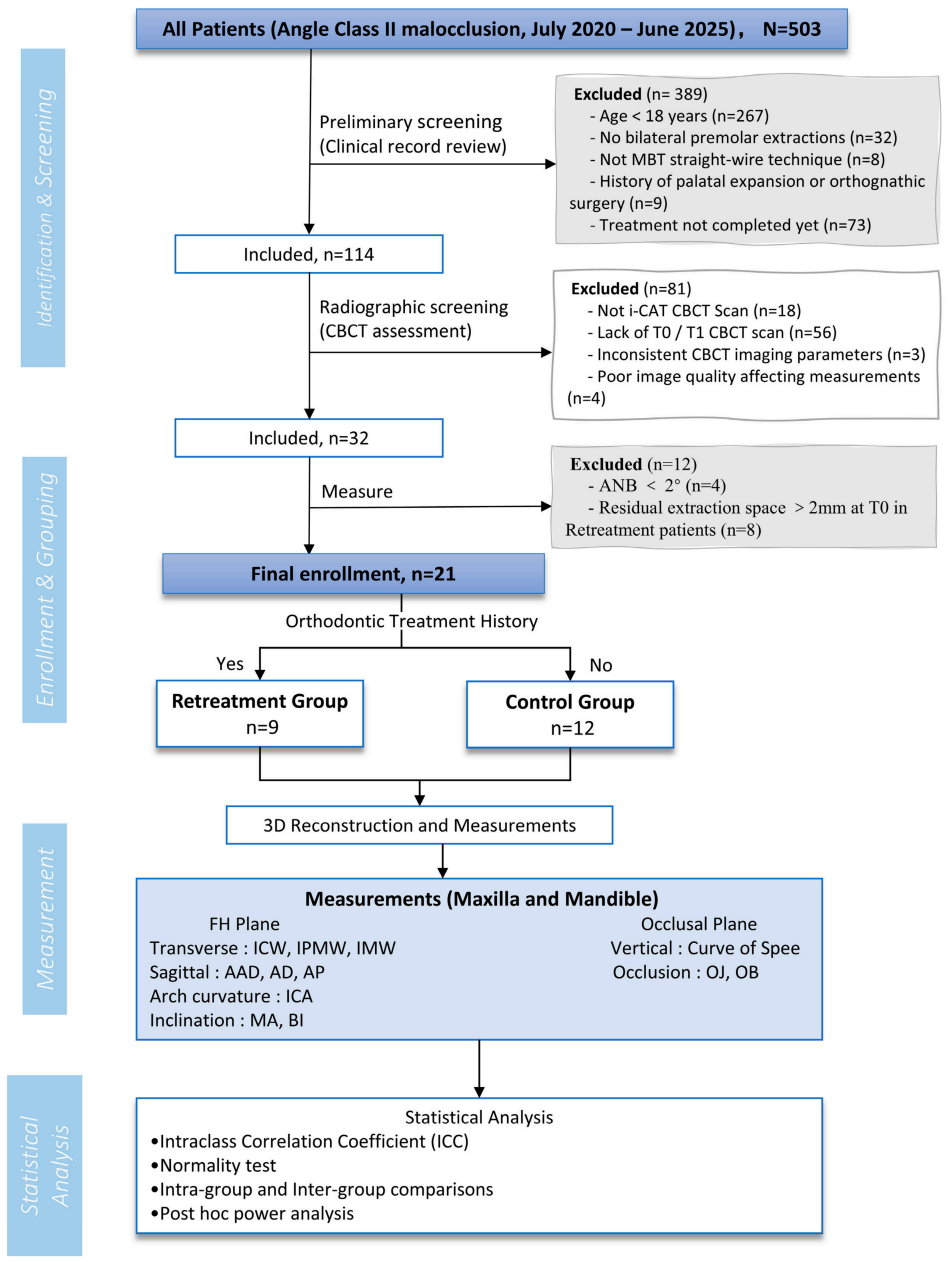
Study flowchart. The study was executed through four integrated phases: (1) identification and screening of subjects; (2) enrollment and allocation; (3) 3D reconstruction and multi-dimensional measurement of arch and dental variables; and (4) statistical analysis. Abbreviations: CBCT: cone-beam computed tomography; *T*0: pre-treatment; *T*1: post-treatment; ANB: Point A–Nasion–Point B angle; FH plane: Frankfort horizontal plane; OP: occlusal plane; ICW: inter-canine width; IPMW: inter-premolar width; IMW: inter-molar width; AAD: anterior arch depth; AD: arch depth; AP: arch perimeter; ICA: inter-canine angle; MA: mesiodistal angulation; BI: buccolingual inclination; OJ: overjet; OB: overbite; and ICC: Intraclass Correlation Coefficient.

This is an exploratory retrospective cohort study, following the Strengthening the Reporting of Observational Studies in Epidemiology (STROBE) guidelines. The protocol received approval from the Ethics Committee of College & Hospital of Stomatology, Guangxi Medical University (Approval No. 2025054). All procedures were conducted in accordance with the Declaration of Helsinki. The overall research workflow is illustrated in Figure 1.

### 2.2. Participants and Eligibility Criteria

Patients were consecutively screened from a cohort treated by a single orthodontic specialist between July 2020 and June 2025 at the Department of Orthodontics, College & Hospital of Stomatology, Guangxi Medical University.

The inclusion criteria were:

(1) Angle Class II malocclusion treated with fixed straight-wire appliances and bilateral premolar extractions (extraction of four first or second premolars). For the retreatment patients, extractions must have been performed during the prior orthodontic treatment with residual spaces <2 mm per site; for the first-time control patients, extractions were performed during the current treatment.
(2) ANB angle >2°.
(3) Age ≥18 years.
(4) High-quality CBCT images obtained at both the beginning (*T*0) and completion (*T*1) of the current treatment using a single i-CAT scanner (Imaging Sciences International, Hatfield, PA, USA).

The exclusion criteria were:

(1) History of palatal expansion (e.g., RME or MARPE) or functional appliance therapy (e.g., Twin-block or Frankel II).
(2) Missing permanent molars (excluding third molars).
(3) Presence of extensive prosthetic restorations, dental implants, or severe periodontitis.
(4) Previous orthognathic surgery, craniofacial syndromes, severe skeletal deformities, or systemic diseases affecting bone metabolism.
(5) Incomplete or discontinued treatment.

Following a consecutive enrollment process, 21 female subjects were enrolled and allocated into two groups based on their history of prior orthodontic intervention: the Retreatment Group (*n* = 9), comprising patients with skeletal and dental Class II malocclusion and a history of prior orthodontic treatment (i.e., initial treatment) involving the extraction of four premolars, and the Control Group (*n* = 12), consisting of untreated Class II patients undergoing four-premolar extraction therapy for the first time, with no history of prior orthodontic intervention. This observed sex distribution was a consequence of the consecutive enrollment process.

All patients were treated with a self-ligating bracket system (Damon™ Q, Ormco Corporation, Orange, CA, USA) featuring a 0.022 × 0.028-inch slot, programmed with the MBT™ prescription (incorporating built-in standard tip and torque values to guide three-dimensional tooth positioning). The archwire sequence progressed from 0.012-inch round nickel-titanium (NiTi) wire for initial leveling and alignment, to a 0.016 × 0.022-inch rectangular NiTi wire, and finally to a 0.019 × 0.025-inch stainless steel wire for space closure and detailing. Reverse curve of Spee archwires (0.019 × 0.025-inch stainless steel wire) were incorporated as needed to level and align the dental arches and control vertical dimensions. Space closure was achieved using sliding mechanics on the 0.019 × 0.025-inch stainless steel wire with active tie-backs and elastomeric chains delivering a force of approximately 100 g per side. Intermaxillary Class II elastics (Ormco, Corporation, Orange, CA, USA) were utilized conservatively during the space-closure and finishing stages to assist in sagittal correction and molar relationship detailing while minimizing vertical side effects. Temporary anchorage devices (TADs, diameter: 1.6 mm; length: 10 mm; Cibei Medical Instruments, Ningbo, China) were strategically placed bilaterally at the maxillary infrazygomatic crest in six patients within the Retreatment Group to provide absolute skeletal anchorage and facilitate full-arch distalization during retraction.

### 2.3. CBCT Data Acquisition and 3D Reconstruction

CBCT data were acquired using a single i-CAT scanner (120 kV, 7 mA, and a 0.25 mm axial slice thickness) with patients in a natural head position and maximum intercuspation. The DICOM files were imported into Mimics (v21.0, Materialise, Leuven, Belgium) for the segmentation of maxillary and mandibular dental and skeletal structures. Three-dimensional (3D) surface models were subsequently reconstructed and exported in STL format for multi-dimensional analysis.

### 2.4. Reference Planes and Variable Measurements

#### 2.4.1. Landmark Identification

Table 1 and Table 2, respectively, describe the definitions of landmarks and measurement variables. Landmark identification was performed on 3D digital models and confirmed in orthogonal views (axial, coronal, and sagittal). To standardize comparisons across patients with different extraction patterns, the “target premolar” was consistently defined as the tooth positioned immediately anterior to the first permanent molar.

#### 2.4.2. Standardized Reference Planes

**Figure 2:**
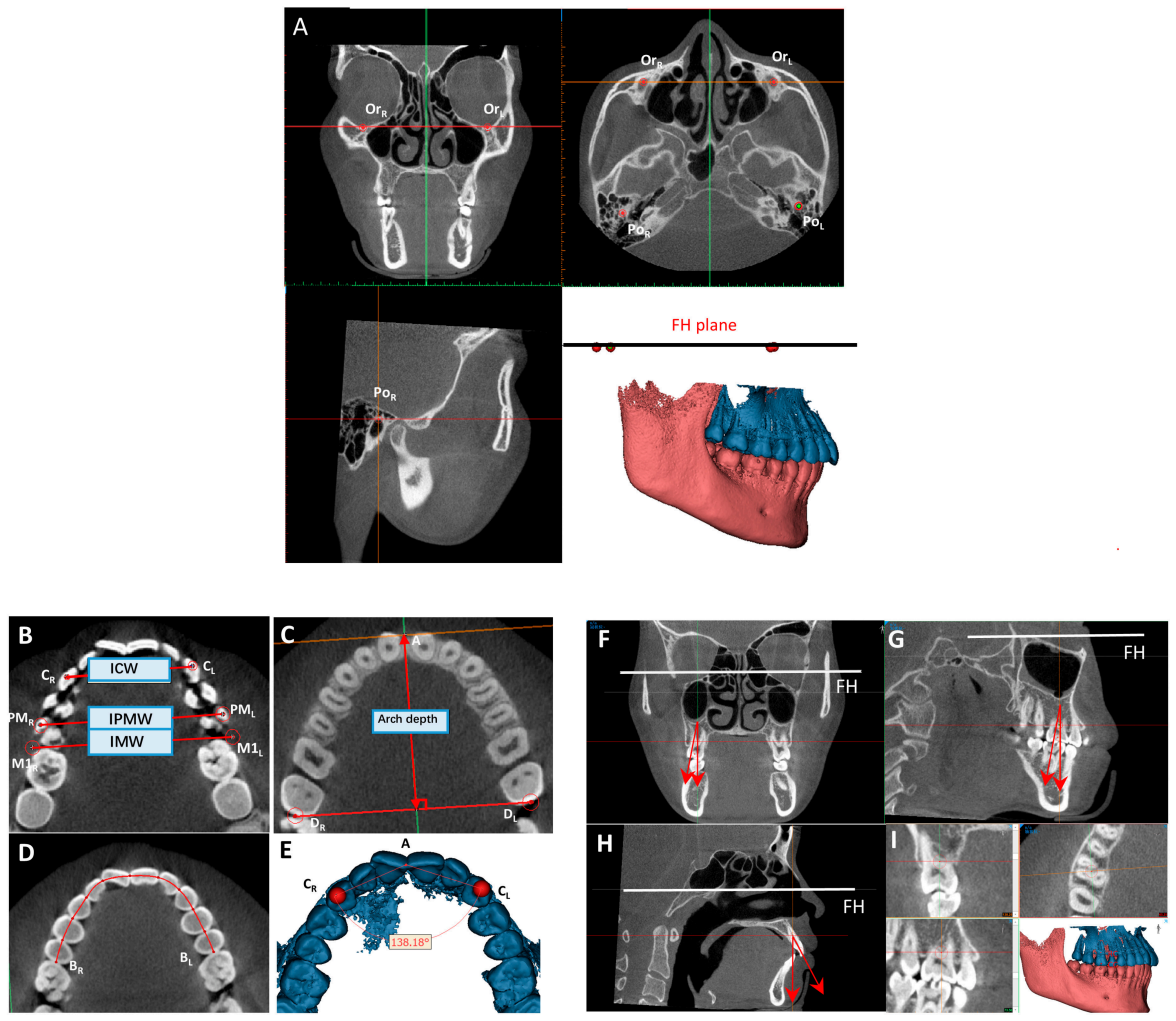
(**A**) Reorientation of the Frankfort horizontal plane (FH plane). Localization of the landmarks Orbitale (Or) and Porion (Po) in orthogonal views; the FH plane was constructed by connecting the bilateral Orbitale (OrL, OrR) and the right Porion (PoR) points. The 3D model was then reoriented using the “create reslice plane” tool to position the FH plane parallel to the horizontal. (**B**) Measurements of intercanine width (ICW), interpremolar width (IPMW), and intermolar width(IMW); (**C**) measurements of arch depth; (**D**) measurements of arch perimeter; (**E**) measurements of intercanine angle; (**F**) buccolingual inclination (BI) measured on the coronal view (molar, premolar, and canine); (**G**) mesiodistal angulation (MA) on the sagittal view (molar, premolar, and canine); (**H**) BI of a central incisor measured on the sagittal view; and (**I**) localization of the root center (RC) and crown center (CC).

**Figure 3:**
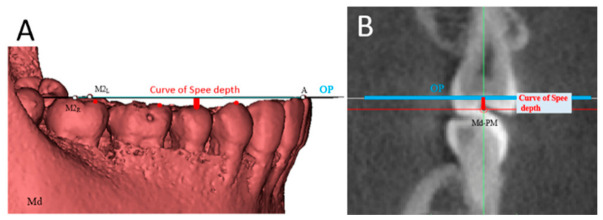
(**A**) Reorientation of the occlusal plane (OP). The OP was established by connecting the landmarks on the bilateral mandibular second molars (M2R, M2L) and the mesial contact point of the central incisor (point A). The 3D model was then reoriented using the “create reslice plane” tool to position the OP parallel to the horizontal. (**B**) Measurement of the depth of the curve of Spee (example shown on a premolar). On the coronal view, the perpendicular distance from a cusp tip to the OP is measured. The depth of the curve of Spee is recorded as the average of the maximum measured distances on the left and right sides.

Two distinct reference planes were utilized to evaluate dental and arch changes:

(1) Frankfort Horizontal Plane (FH Plane): This plane is constructed by connecting the bilateral Orbitale points (Or) and the right Porion (Po) [19]. This plane served as the primary reference for reorienting the CBCT data volume (Figure 2A).
(2) Occlusal Plane (OP): This plane is defined by the midpoint of the mandibular central incisal edges (A) and the distobuccal cusp tips of the bilateral mandibular second molars (M2) [20]. This plane was oriented parallel to the horizontal plane to facilitate vertical and occlusal assessments (Figure 3A).

#### 2.4.3. Variable Measurement

A comprehensive suite of measurements was obtained relative to these reference planes.

FH plane-Based Measurements (Figure 2): This category included transverse widths (intercanine width [ICW], interpremolar width [IPMW], intermolar width [IMW]), sagittal length (anterior arch depth [AAD], total arch depth [AD], and arch perimeter [AP]), intercanine angle, and tooth inclination (mesiodistal angulation [MA], buccolingual inclination [BI]). Notably, tooth inclination was evaluated using the tooth long axis (Table 1). The long axis was defined as the line connecting the crown center and root center, thereby providing a more biologically accurate assessment than the clinical crown axis [18,21,22].

OP-Based Measurements: The depth of the curve of Spee was calculated as the average of the maximum perpendicular distances from the cusp tips of the canines and all posterior teeth to the OP (Figure 3B). Additionally, occlusal parameters including overjet (OJ) and overbite (OB) were measured relative to this plane (Table 2) [23].

#### 2.4.4. Measurement Reliability

All landmark identifications and measurements were performed by a single operator. To assess intra-rater reliability, the entire procedure was repeated after a two-week interval. The Intraclass Correlation Coefficient (ICC) values ranged from 0.919 to 0.975, indicating excellent reproducibility for all variables.

### 2.5. Statistical Analysis

Statistical analysis was performed using SPSS (v23.0, IBM Corp., Armonk, NY, USA). Shapiro–Wilk tests were used to confirm the normality of the data. Given that all data met or approximated normal distribution, descriptive statistics are presented as mean ± *SD*. Between-group comparisons were conducted using independent samples *t*-tests, and within-group changes were evaluated with paired samples *t*-tests. The significance level was set at *p* < 0.05 for all statistical tests.

To account for the limited sample size, *Hedges’ g* was calculated as the primary metric for effect size. This provides a bias-corrected estimate that is more robust for small-cohort studies than Cohen’s d. Effect sizes were interpreted using the following benchmarks: small (0.2), medium (0.5), and large (0.8) [24].

## 3. Results

### 3.1. Descriptive Statistics of Baseline Characteristics

The study population consisted of 21 female participants, divided into a Retreatment Group (n = 9) and a Control Group (n = 12). As detailed in Table 3, the two groups were well-matched at baseline, with no statistically significant differences observed in age (*p* = 0.484) or ANB angle (*p* = 0.173).

### 3.2. Comparison of Archform and Occlusal Variables

**Figure 4:**
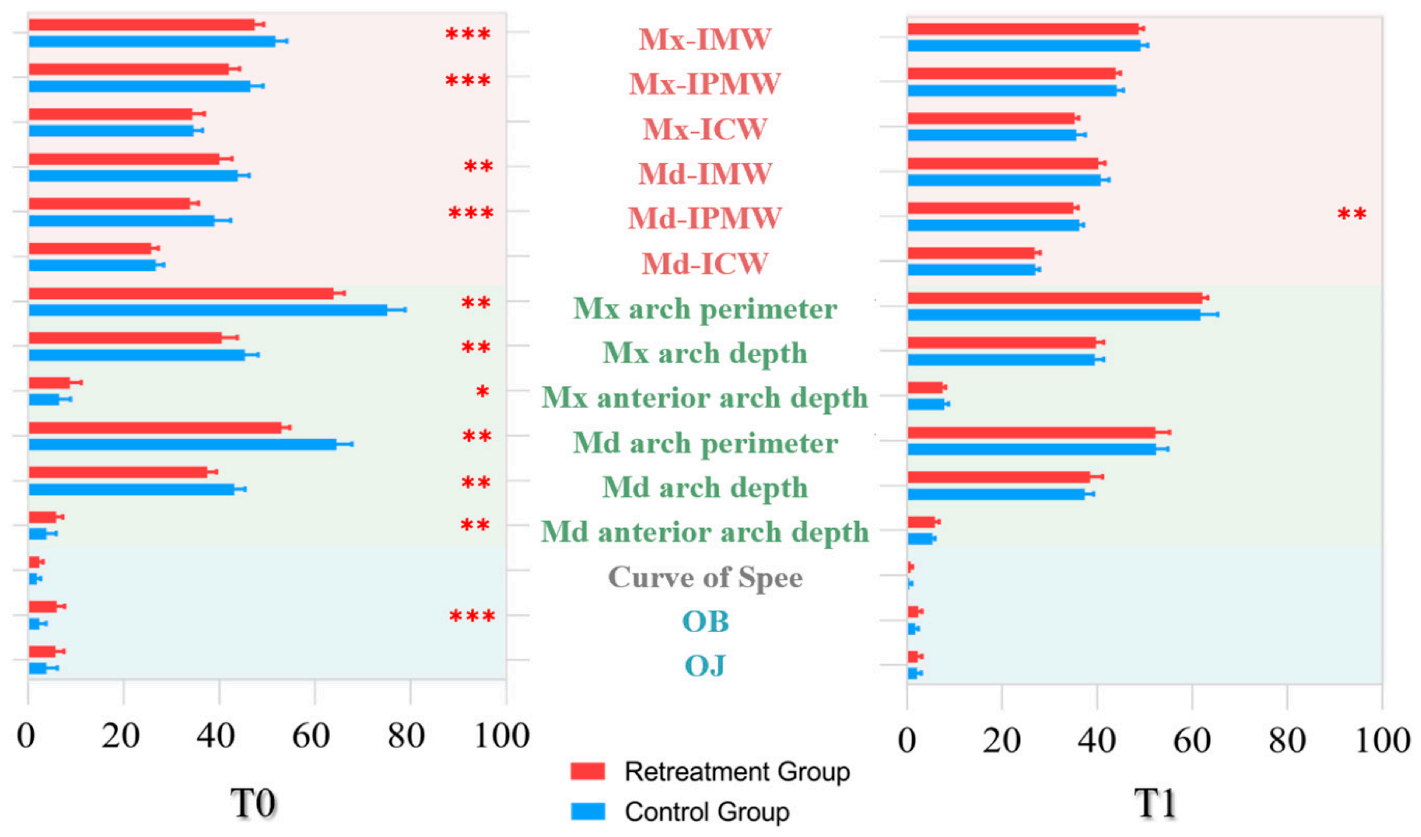
Pre-treatment (*T*0) and post-treatment (*T*1) linear parameter comparisons between the two groups (mm). * *p* < 0.05, ** *p* < 0.01, and *** *p* < 0.001.

**Figure 5:**
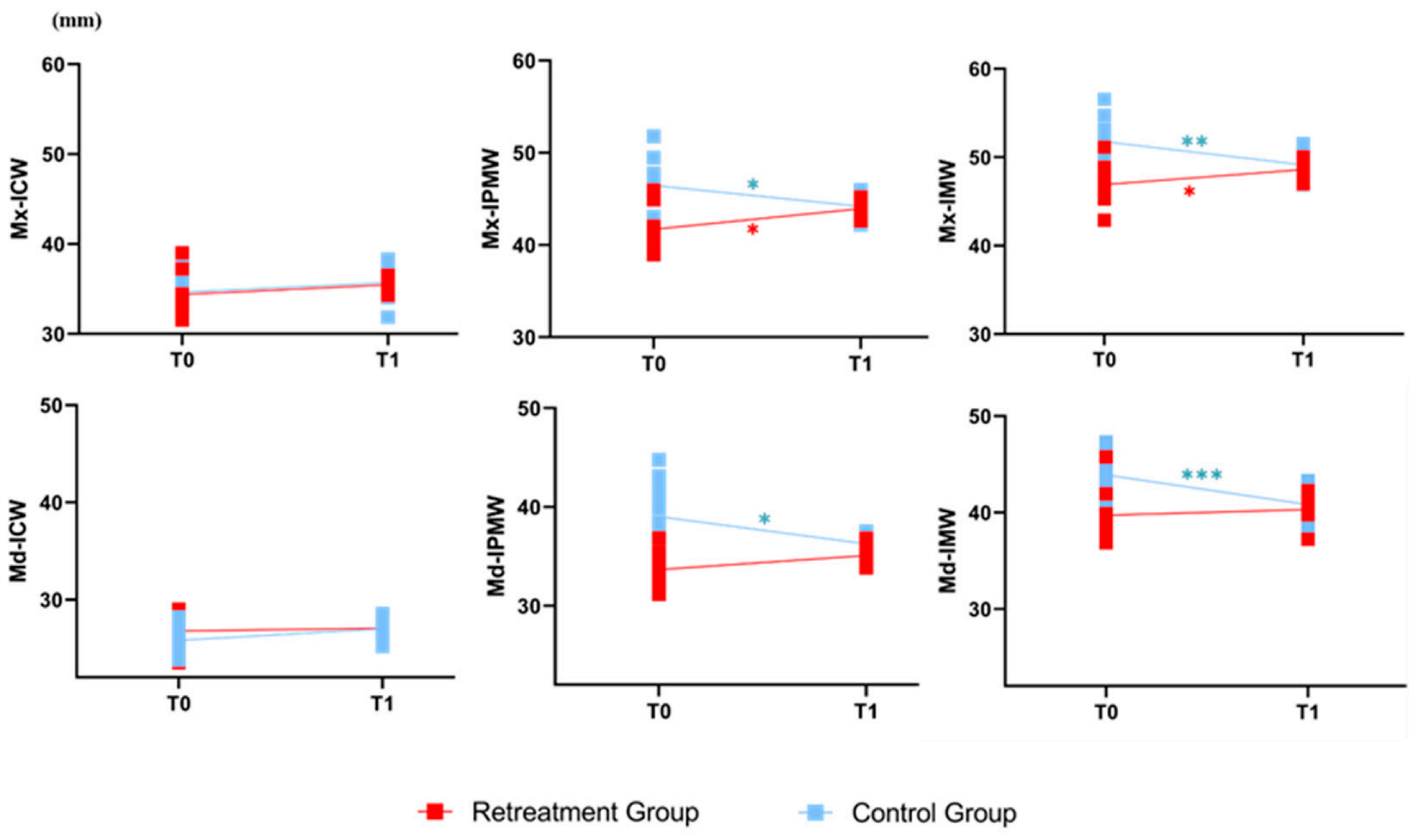
*T*0–*T*1 changes in arch width. Asterisks indicate a statistically significant change from *T*0 to *T*1. * *p* < 0.05, ** *p* < 0.01, and *** *p* < 0.001.

**Figure 6:**
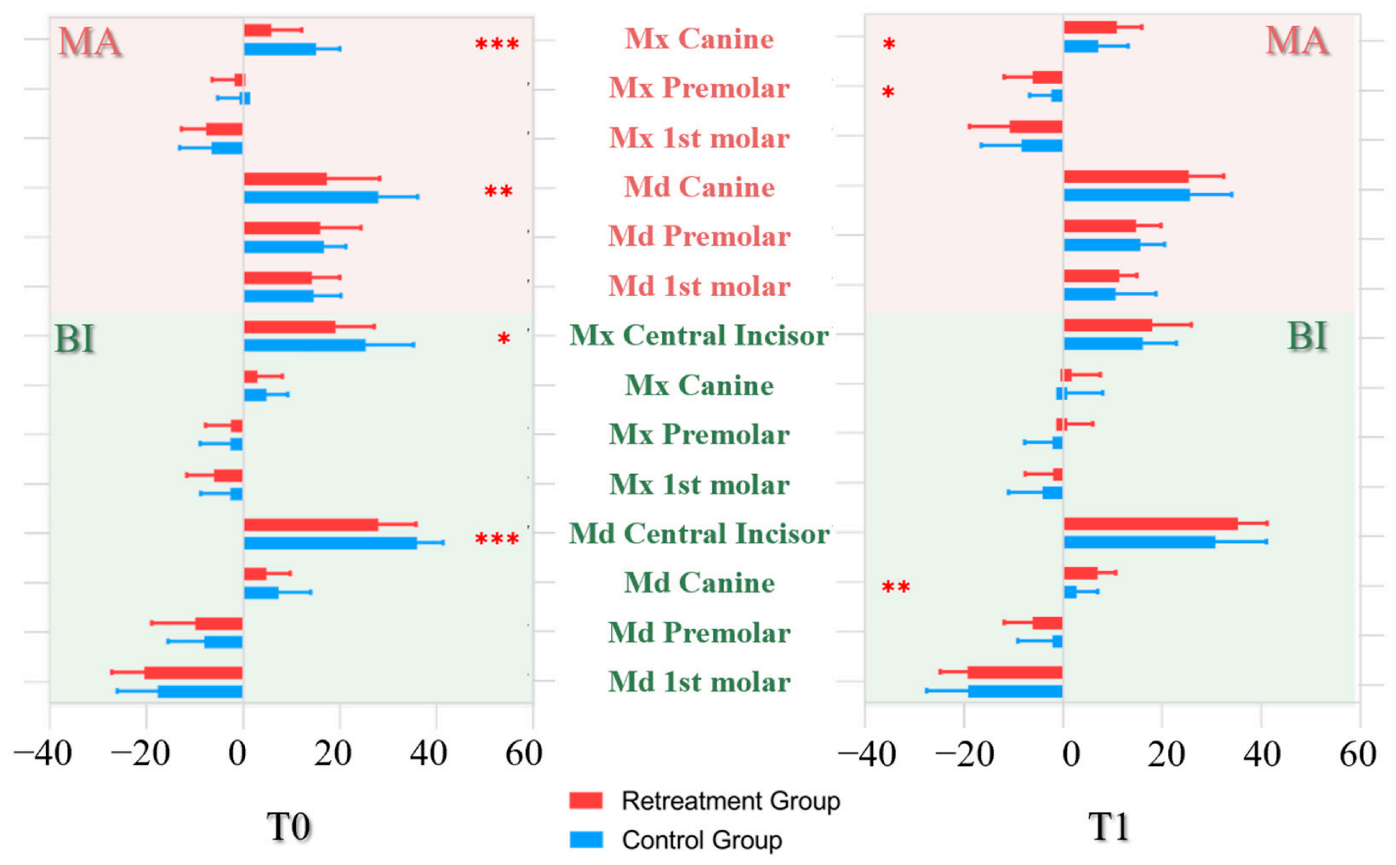
Pre-treatment (*T*0) and post-treatment (*T*1) angle parameter comparisons between the two groups (°). * *p* < 0.05, ** *p* < 0.01, and *** *p* < 0.001.

Comparisons of archform and tooth inclination variables between groups at each stage, as well as changes from *T*0 to *T*1 within the Retreatment Group, are summarized in Table 4 and Table 5. Corresponding graphical presentations of arch linear and angular parameter comparisons are provided in Figure 4, Figure 5 and Figure 6. The detailed findings are described below.

#### 3.2.1. Transverse Width (Table 4, Figure 4)

At *T*0, the Retreatment Group exhibited severe transverse deficiency across both dental arches compared to the Control Group. Specifically, interpremolar width (IPMW) was significantly narrower in the Retreatment Group by 4.90 mm in the maxilla (*p* < 0.001, *Hedges’ g* = 1.88) and 5.37 mm in the mandible (*p* < 0.001, *Hedges’ g* = 1.82). Similarly, intermolar width (IMW) values in the maxilla and mandible were significantly reduced by 4.85 mm and 3.80 mm, respectively (*p* ≤ 0.002, *Hedges’ g* > 1.50). No significant differences in intercanine width (ICW) were observed between groups at baseline.

During treatment (*T*0–*T*1), opposing trends were identified: the Retreatment Group achieved a significant increase in maxillary IPMW (+2.22 mm, *p* = 0.028, *Hedges’ g* = 0.81) and IMW (+1.69 mm, *p* = 0.021, *Hedges’ g* = 0.86). In the mandibular arch, IPMW also showed an increasing trend that was not statistically significant but demonstrated a moderate effect size (Md IPMW +1.41 mm, *p* = 0.062, *Hedges’ g* = 0.65). In contrast, the Control Group exhibited a significant reduction in both the maxilla and mandible, with width decreases ranging from 2.42 mm to 3.07 mm (*p* < 0.05, *Hedges’ g* = 0.68–1.36). ICW remained stable in both groups throughout the treatment period (Figure 5).

By *T*1, maxillary widths in the Retreatment Group had been corrected to levels comparable with those of the Control Group (*p* > 0.05). However, the Retreatment Group maintained a significantly narrower mandibular IPMW, with a mean difference of −1.20 mm (*p* = 0.008, *Hedges’ g* = 1.11), indicating that complete transverse recovery was achieved in the maxilla but remained limited in the mandible.

#### 3.2.2. Sagittal Length (Table 4, Figure 4)

At *T*0, the Retreatment Group showed a unique sagittal pattern: despite significantly smaller total arch depth (AD) and perimeter (AP) from prior extractions, their anterior arch depth (AAD) was significantly larger than that of the Control Group (Mx: +2.35 mm, *p* = 0.032, *Hedges’ g* = 0.98; Md: +1.92 mm, *p* = 0.017, *Hedges’ g* > 1).

During treatment (*T*0–*T*1), AAD tended to increase in the Control Group (Mx: +1.40 mm, *p* = 0.068, *Hedges’ g* = 0.59; Md: +1.39 mm, *p* = 0.049, *Hedges’ g* = 0.69), while the Retreatment Group achieved a significant reduction in Mx-AP (−1.92 mm, *p* = 0.020, *Hedges’ g* = 0.87) and a decreasing trend in Mx-AAD (−1.24 mm, *p* = 0.084, *Hedges’ g* = 0.69).

By *T*1, no statistically significant differences were observed between the two groups with respect to AAD, AD, or AP.

#### 3.2.3. Arch Curvature (Table 4, Figure 6)

At *T*0, the Retreatment Group exhibited significantly smaller intercanine angles (ICAs) in both arches (*p* ≤ 0.01), which is indicative of a tapered anterior arch form.

During treatment (*T*0–*T*1), opposing trends were observed: the Retreatment Group achieved a significant increase in Mx-ICA (+8.67°, *p* = 0.011) but only a non-significant increase in Md-ICA (+3.95°, *p* = 0.159), while the Control Group showed a decreasing trend in both arches (−7.72° to −7.88°, *p* ≥ 0.086).

By *T*1, the maxillary ICA difference between the two groups was resolved (*p* = 0.528), whereas the mandibular ICA in the Retreatment Group remained significantly smaller than that in the Control Group (*p* = 0.037, *Hedges’ g* = 1.02).

#### 3.2.4. Vertical and Occlusal Parameters (Table 4, Figure 4)

The depth of the curve of Spee showed no significant difference between the two groups at *T*0 (*p* = 0.102) or *T*1 (*p* = 0.211). Both groups achieved a statistically significant reduction in depth after treatment, with a decrease of 1.58 mm in the Retreatment Group and 1.27 mm in the Control Group (both *p* < 0.001).

Regarding occlusal parameters at *T*0, the Retreatment Group presented a clinical deep overjet (5.73 mm, *p* = 0.055, *Hedges’ g* = 0.87) and a significantly greater overbite (5.85 mm, *p* < 0.001, *Hedges’ g* = 2.24) relative to the Control Group. By *T*1, both overjet and overbite in the Retreatment Group were corrected to levels comparable to those of the Control Group.

#### 3.2.5. Tooth Inclination (Table 5, Figure 6)

At *T*0, the Retreatment Group showed smaller mesiodistal angulation (MA) and buccolingual inclination (BI), indicating significant distal crown tipping of canines (*p* ≤ 0.001) and lingual inclination of central incisors (*p* < 0.05), respectively.

During treatment (*T*0–*T*1), the Retreatment Group underwent primary compensatory adjustments. For MA, significant changes occurred in most teeth, characterized by distal root tipping (increased MA) of the canines (Mx Canine + 4.95°, *p* = 0.010, *Hedges’ g* = 0.65; Md Canine + 8.06°, *p* < 0.001, *Hedges’ g* = 0.99) and mesial root tipping (decreased MA) of the maxillary premolars (−4.29°, *p* = 0.002, *Hedges’ g* = 0.84), maxillary first molars (−3.08°, *p* = 0.306, *Hedges’ g* = 0.51), and mandibular first molars (−2.84°, *p* = 0.040, *Hedges’ g* = 0.50). For BI, all significant changes reflected buccal tipping to resolve initial compensations. Specifically, the mandibular central incisor BI increased by 7.28° (*p* < 0.001, *Hedges’ g* = 1.52), while the mandibular premolars showed a 3.78° reduction in lingual inclination (from −9.81± 8.80° to −6.03 ± 5.80°, *p* = 0.009, Hedges’g = 0.67). The maxillary premolars and first molars also tipped buccally by 3.39° and 3.98°, respectively (*p* < 0.01, *Hedges’ g* range: 0.68–0.78).

By *T*1, most inclination differences between groups were resolved. However, residual patterns persisted in the Retreatment Group, including greater mesial root tipping of maxillary premolars (mean difference = −3.74°, *p* = 0.023) and greater labial inclination of mandibular canines (mean difference = +4.17°, *p* = 0.002) compared to the Control Group.

### 3.3. Representative Case

**Figure 7:**
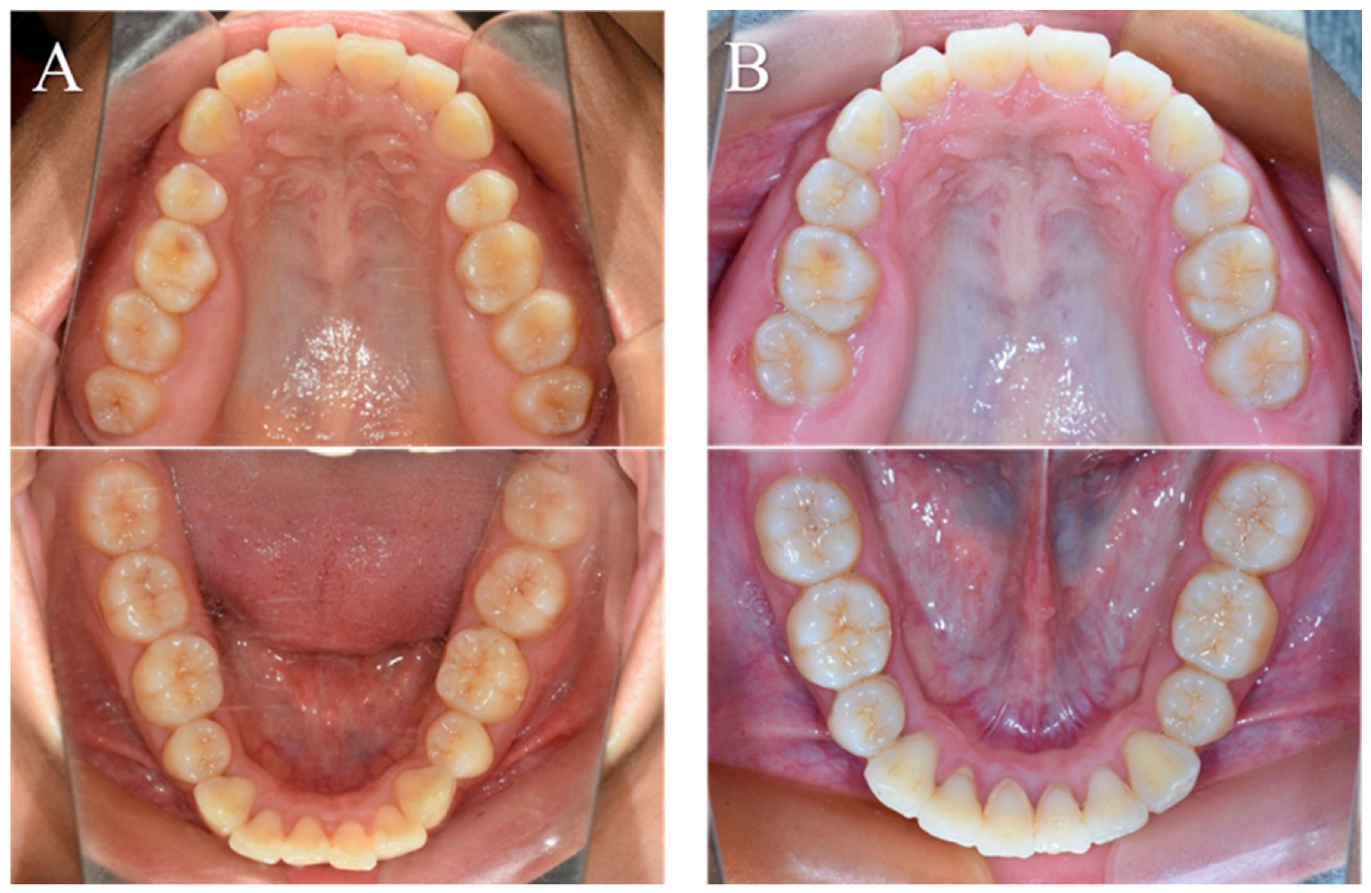
Arch form changes in the Retreatment Group in a typical case. (**A**) Pre-treatment; (**B**) post-treatment.

A representative case illustrating the arch form changes in the Retreatment Group is shown in Figure 7. Pre-treatment and post-treatment arch forms are presented, demonstrating the typical morphological changes observed in this group.

## 4. Discussion

### 4.1. Methodological Considerations

The sex distribution in this study was an incidental result of the patient selection process and aligns with adult retreatment demographics [1,3], reflecting the clinical reality that females are the primary group seeking secondary intervention. Although cohort size was limited by rigorous inclusion criteria, the primary outcomes demonstrated substantial effect sizes (*Hedges’ g*: 1.55–1.94). As a bias-corrected estimate suited for small cohorts, *Hedges’ g* values exceeding the 0.80 threshold for a “large” effect indicate that the structural differences between groups are sufficiently robust to overcome potential limitations in statistical power.

Dual reference planes were utilized to distinguish absolute tooth positions from relative occlusal changes, ensuring reliability [17,25]. While functional parameters were evaluated relative to the OP [26], this plane is inherently susceptible to treatment-induced changes. Consequently, BI and MA were measured relative to the stable FH plane, which provides a more robust anatomical reference for evaluating true dental inclination [27].

CBCT enhanced measurement accuracy by enabling evaluation based on the tooth’s true long axis, defined as the line connecting the crown center to the root center [28], rather than the clinical crown axis [29]. This approach minimizes interference from apical curvature and provides a more accurate measure of tooth orientation within the alveolar bone.

### 4.2. Characteristics of Dental Arch Morphology at T0

At *T*0, the Retreatment Group exhibited significant malocclusions in all three dimensions, with transverse narrowing being the most prominent feature. Specifically, interpremolar width (IPMW) and intermolar width (IMW) were 4.17–5.37 mm narrower than in controls, with the most pronounced constriction in the premolar region (the area most adjacent to extraction sites). While previous studies have reported an average reduction of approximately 2 mm in dental arch width during extraction orthodontics due to mesial movement of posterior teeth into narrower basal bone [5,30,31], the magnitude of narrowing observed in this study substantially exceeds this expected therapeutic range.

This excessive narrowing likely resulted from abnormal lingual movement of teeth adjacent to extraction sites, attributable to suboptimal biomechanics during initial treatment. This interpretation is supported by simulation studies. A finite element analysis by Sayegh et al. [32] on extraction patients treated with self-ligating brackets demonstrated that during space closure with sliding mechanics, significant lingual tipping of molars occurred when using smaller-dimension archwires (0.017” × 0.025” and 0.018” × 0.025”), whereas a stiffer archwire (0.019” × 0.025”) prevented significant tipping. Similarly, an in vitro study by Zhu et al. [33] identified a comparable lingual tipping effect in clear aligner therapy, attributed to the “bowing effect,” an elastic deformation of the aligner spanning the extraction site that generates an unwanted inward force on posterior teeth.

In our study, the Retreatment Group exhibited a greater tendency toward lingual inclination than controls in maxillary molars, mandibular molars, and mandibular premolars. However, these differences were not statistically significant and demonstrated small-to-medium effect sizes (range: 0.23–0.56), suggesting that this trend still requires verification with a larger sample size and should not be regarded as a definite conclusion at present.

This transverse discrepancy was accompanied by compensatory abnormalities in the sagittal and vertical dimensions. The Retreatment Group presented with increased overjet (5.73 mm) and overbite (5.85 mm), accompanied by lingual incisor tipping and excessive distal tipping of canine crowns, indicating a loss of torque and tip control. Of high diagnostic significance, the Retreatment Group exhibited paradoxically greater maxillary anterior arch depth (AAD) despite smaller total arch depth (AD) due to previous extractions. This feature, corroborated by increased overjet, indicates that extraction spaces were ineffectively used for anterior retraction due to anchorage loss, a key factor in initial treatment failure [34]. The biomechanical basis is supported by evidence that significant mesial movement of first molars (1.92–3.99 mm) occurs during space closure, even with skeletal anchorage [29]. Such mesial tipping serves as a definitive marker of anchorage loss and is more likely in Class II patients undergoing maxillary premolar extractions, a patient profile that directly matches our study population [35].

Anterior torque loss and uncontrolled canine tipping are pathognomonic of the iatrogenic “roller-coaster effect” during space closure. Attia et al. [29] indicate that friction mechanics can cause uncontrolled tipping or rotation of anterior teeth, necessitating specific biomechanical designs (e.g., varying hook heights or archwire bends) to prevent torque loss during retraction. This sagittal torque loss directly compromises vertical stability; inadequate torque triggers incisor extrusion, deepening the curve of Spee and the overbite [36]. Xiang et al. [37] validated this cascade (torque loss, extrusion, and posterior mesial tipping) using 3D finite element analysis. This vertical and sagittal decompensation parallels the vertical bowing effect in Class II lingual orthodontics [38]. Because palatal traction forces pass further from the center of resistance, lingual mechanics inherently induce greater anterior torque loss and extrusion during retraction, as confirmed by finite element analyses [39,40]. Counteracting these effects requires biomechanical compensations like temporary skeletal anchorage devices [41]. The baseline morphology of low torque, deep overbite, and marked lingual tipping in our retreatment cohort mirrors the long-term manifestation of such uncontrolled side effects during initial space closure.

Collectively, in contrast to untreated individuals, Angle Class II patients undergoing orthodontic retreatment with a history of four-premolar extraction exhibit a distinct arch collapse morphology. As defined in this study, arch collapse refers to the dentoalveolar phenotype characterized by transverse narrowing (especially in the premolar region), which is often accompanied by compensatory anterior elongation, loss of incisor torque, and abnormal canine inclination, all of which collectively increase the overbite and overjet. It should be noted that the present data primarily reflect changes in tooth position and inclination; whether skeletal collapse is also involved requires further imaging validation.

### 4.3. Treatment Changes from T0 to T1

The main treatment changes in the Retreatment Group during *T*0–*T*1 involved transverse arch width, mesiodistal angulation (MA), and buccolingual inclination (BI). The discussion therefore focuses on these key outcomes.

Arch width changes diverged between groups: expansion in the Retreatment Group versus constriction in controls (Figure 5). A critical interpretative distinction must be made regarding the transverse dental arch width changes between the two cohorts. In the Control Group, both arches exhibited significant, moderate narrowing (approximately 2 mm on average) during space closure. This narrowing represents a planned, physiological dentoalveolar adaptation as the posterior teeth migrate mesially into the naturally narrower anterior segment of the basal bone arch [5,30,31,42]. Thus, this narrowing should not be interpreted as treatment deterioration or an iatrogenic side effect. In contrast, the Retreatment Group presented at *T*0 with pathological, iatrogenic constriction (4.17–5.37 mm narrowing) that far exceeded this expected physiological range. Consequently, the posterior dental expansion achieved during retreatment (maxillary IMPW: 2.22 mm, *p* = 0.028, *Hedges’ g* = 0.81; IMW: 1.69 mm, *p* = 0.021, *Hedges’ g* = 0.86) was strictly corrective, utilizing skeletal anchorage to upright the previously collapsed segments and restore the dental arch to its stable, normal physiological width. The mandibular arch width remained stable.

This maxillary expansion is attributed to two factors. First, in six of nine retreatment patients, maxillary TADs were employed to facilitate full-arch distalization, repositioning posterior teeth into broader segments of the basal bone. This biomechanical effect is supported by Song et al. [43], who reported a 0.93 mm increase in molar arch width following TAD-assisted distalization, which is consistent with our findings. Second, absolute anchorage from TADs may have allowed full expression of the self-ligating system’s expansion potential, manifested as increased buccal crown torque. Lineberger et al. [44,45] demonstrated that in passive self-ligating systems, maxillary premolar expansion (2.0–2.2 mm) was accompanied by a significant increase in buccal crown torque (mean 5.5°). Similarly, Lucchese et al. [45] reported that arch expansion with self-ligating appliances is proportional to torque increases. In the present study, maxillary premolars and first molars in the Retreatment Group exhibited significant buccal tipping, with mean inclination increases of 3.39° and 3.98°, respectively (*p* < 0.01, *Hedges’ g* = 0.68–0.78).

The mandibular posterior arch remained stable throughout retreatment (*p* > 0.05), in accordance with the pre-established therapeutic objective. This outcome resulted directly from the anchorage strategy, in which the mesially displaced mandibular first molars served as a stable anchorage unit to achieve a Class I occlusal relationship. Specifically, IMW showed minimal change (mean 0.58 mm; *Hedges’ g* = 0.21). Although IPMW increased by 1.41 mm without reaching statistical significance (*p* = 0.062), it demonstrated a medium effect size (*Hedges’ g* = 0.65), suggesting a clinically relevant trend that may be undetected due to limited statistical power.

This limited change likely reflects mandibular anatomical constraints, which permit only minor dentoalveolar changes [46,47]. As Andrews suggested, the WALA ridge serves as a key anatomical boundary for the alveolar process, and respecting such boundaries is critical for ensuring long-term stability [48,49].

Intercanine width remained stable in both groups, aligning with the principle of preserving this dimension to reduce relapse risk. Housley et al. [50,51] showed that expanded intercanine widths tend to relapse post-retention. Finite element analysis by Cai et al. [51] revealed high stress concentrations at the canine cervical and apical regions during buccal expansion, increasing risks of bone dehiscence and relapse. Maintaining intercanine width thus avoids adverse effects and supports stability. The intercanine angle (ICA) increased, indicating a transition from a sharp to a more rounded, functional arch curve, while arch depth and circumference showed no significant changes.

Overall, improvements in arch form and occlusion were achieved through precise three-dimensional adjustments in dental inclination (MA and BI), enabled by maxillary transverse expansion. Key corrective movements included distal uprighting of the canine roots and labial tipping of the mandibular incisors. This combination effectively reversed the abnormal morphology associated with the “roller coaster effect”, which in turn leveled the curve of Spee and significantly improved overbite and overjet. The correction was transverse-led, supported by maxillary distalization when needed, allowing sagittal and vertical correction via fine-tuned dental positioning.

### 4.4. Treatment Outcomes at T1

By *T*1, overjet and overbite were within normal limits, and most arch dimensions did not differ significantly between groups, indicating that retreatment achieved outcomes comparable to the Control Group.

Despite similar results, some differences persisted due to prior treatment limitations. The Retreatment Group exhibited narrower mandibular premolar width, which is likely attributable to anatomical constraints and limited space availability. This finding aligns with the inherent limitations of mandibular basal bone, which offers less capacity for dentoalveolar expansion than the maxilla. The excessive labial canine inclination and tapered arch form observed at *T*1 appear compensatory, enabling anterior alignment within the confined space and cortical boundaries of the mandibular arch. Mesial root tipping of maxillary first premolars likely resulted from suboptimal force vectors during TAD-assisted distalization, where forces did not pass through the center of resistance. This finding underscores the technical sensitivity of these mechanics and the importance of precise force vector control.

Given the anatomical limitations in mandibular premolar width recovery and the well-documented tendency for mandibular arch width to gradually decrease both post-retention and during the natural aging process [52,53], a targeted retention protocol is required to mitigate the re-narrowing of the lower IPMW. Retrospective studies indicate that longer retention periods yield better stability for cases involving significant treatment changes, particularly in the mandible [54]. However, systematic reviews confirm that high-quality evidence supporting the clinical superiority of one specific retainer type or duration remains insufficient [55]. Consequently, a conventional canine-to-canine fixed retainer is biomechanically inadequate for maintaining transverse posterior dimensions, leading empirical clinical practice to suggest a dual-retention strategy. A pragmatic approach involves combining a fixed lingual retainer with a full-coverage removable appliance specifically designed to control transverse dimensions [56]. In cases exhibiting severe transverse instability, extending the fixed retainer to the premolars may be considered [57,58]. Ultimately, definitive and evidence-based guidelines for this specific phenotype still require further large-scale investigation.

### 4.5. Limitations and Future Directions

This study has several limitations. First, the retrospective design may introduce selection bias despite consecutive enrollment. Second, the sample size is small, though it represents the maximum eligible cases meeting the rigorous inclusion criteria for this specific “four-premolar extraction retreatment” phenotype. Third, the exclusive inclusion of female participants, while reflecting the primary demographic for adult retreatment, limits generalizability to males. Fourth, TAD use varied within the Retreatment Group (six of nine patients), introducing treatment heterogeneity; this reflects real-world clinical decisions based on individual anatomical and biomechanical needs. Fifth, the lack of post-retention follow-up data precludes assessment of long-term stability. Thus, the observed changes should be viewed as immediate outcomes, not markers of long-term post-retention stability.

Despite these constraints, the primary outcomes demonstrated statistically significant differences with large effect sizes (*Hedges’ g* > 0.8), underscoring the robustness of the observed structural differences. We also recognize that certain non-significant findings (e.g., lingual inclination tendencies in the Retreatment Group, IPMW change in the mandible) exhibited small-to-medium effect sizes, implying potential Type II errors; these variables might have reached statistical significance with a larger cohort.

This study serves as an exploratory foundation, providing essential baseline effect size estimates for future research. As the demand for adult orthodontic retreatment grows, these preliminary findings can guide the design of large-scale, multicenter prospective studies that include longer follow-up periods and gender-diverse populations to validate the observed outcomes.

## 5. Conclusions

Within the limitations of this preliminary retrospective study, the following observations are made as hypothesis-generating findings rather than definitive conclusions.

(1) Compared to the first-time patients, Angle Class II orthodontic retreatment patients with a four-premolar extraction history present a unique “arch collapse” morphology, characterized by significantly narrower posterior arch widths (4.17–5.37 mm), along with an elongated and tapered anterior arch and abnormal tooth inclination.
(2) During *T*0–*T*1, the Retreatment Group showed maxillary posterior expansion, whereas mandibular arch width did not change significantly. Statistically significant changes were also observed in mesiodistal angulation and buccolingual inclination for most teeth.
(3) At *T*1, most arch dimensions and occlusal parameters were comparable between groups. Nevertheless, mandibular interpremolar width remained narrower in the Retreatment Group, suggesting that complete transverse recovery may be anatomically constrained.
(4) These findings characterize the structural deviations of this phenotype. By providing baseline morphological data and effect size estimates, this study offers an exploratory foundation to inform power calculations for future large-scale, prospective, and multicenter research.

## Figures and Tables

**Table 1:** Definitions of landmarks and reference planes.

Variable	Landmark	Definition
Canine Cusp Tip	C	Most apical point of the canine cusp; if worn, estimated by the intersection of anatomical ridges.
Premolar Buccal Cusp Tip	PM	Most apical point of the premolar buccal cusp; center of wear facet if worn. *
Molar Mesiobuccal Cusp Tip	M	Most apical point of the molar mesiobuccal cusp; center of wear facet if worn.
Mesial Contact Point of the Central Incisor	A	Highest point of the mesial contact of the central incisor.
Mesial Contact Point of the First Molar	B	Highest point of the mesial contact area with the adjacent tooth.
Distal Contact Point of the Second Molar	D	Highest point of the distal contact area with the adjacent tooth.
Orbitale	Or	Lowest point on the inferior orbital margin.
Porion	Po	Most superior point on the external auditory meatus.
Crown Center	CC	The center of the crown, defined by the intersection of three planes bisecting the crown’s buccolingual, mesiodistal, and occluso-gingival diameters.
Root Center	RC	For single-rooted teeth, the midpoint of the root at 1/2 to 2/3 of its length. For multi-rooted teeth, the center of the furcation.
Tooth Long Axis	Axis	Line connecting the crown center and root center.
Frankfort Horizontal Plane	FH plane	Plane through left and right Orbitale and right Porion (left Porion used if right is unclear).
Occlusal Plane	OP	Plane through mesiobuccal cusp tips of mandibular second molars and mesial contact point of mandibular central incisors.

* For standardization in this study, the premolar was defined as the one located immediately anterior to the first permanent molar.

**Table 2:** Definitions of variable measurements.

Category	Reference Plane	Variable	Definition
Transverse Width Measurements	FH Plane	Intercanine Width (ICW)	Distance between the cusp tips of the canines.
		Interpremolar Width (IPMW)	Distance between the buccal cusp tips of the premolars (defined as the premolar immediately anterior to the first permanent molar).
		Intermolar Width (IMW)	Distance between the mesio-occlusal cusp tips of the maxillary or mandibular first permanent molars.
Sagittal Length Measurements		Anterior Arch Depth (AAD)	Perpendicular distance from the mesial contact point of the central incisors to a line connecting the bilateral canine cusp tips.
		Arch Depth (AD)	Perpendicular distance from the mesial contact point of the central incisors to a line connecting the distal contact points of the bilateral second molars.
		Arch Perimeter (AP)	Length of the curve connecting the contact points of all teeth, extending from the mesial contact of the first molar on one side to the contralateral first molar.
Arch Curvature Measurements		Intercanine Angle (ICA)	Angle formed by the bilateral canine cusp tips with the mesial contact point of the central incisors serving as the vertex.
Tooth Inclination Measurements		Buccolingual Inclination (BI)	Premolars/Molars/Canines: Measured on the coronal view as the angle between the tooth long axis projection and a line perpendicular to the FH Plane. Central Incisors: Measured on the sagittal view (sign convention: labial/buccal crown inclination = positive (+); palatal/lingual = negative (−)).
		Mesiodistal Angulation (MA)	Premolars/Molars/Canines: Measured on the sagittal view as the angle between the tooth long axis projection and a line perpendicular to the FH Plane. Central Incisors: Measured on the coronal view (sign convention: distal root inclination = positive (+); mesial root = negative (−)).
Vertical Measurements	OP	Depth of the curve of Spee	Average of the maximum perpendicular distances from the cusp tips of canines and all posterior buccal cusps to the occlusal plane (OP) on both right and left sides.
Occlusal Parameters		Overjet (OJ)	Horizontal distance from the most labial surface of the maxillary central incisor to the incisal edge of the most labial mandibular central incisor.
		Overbite (OB)	Vertical distance between the incisal edges of the maxillary and mandibular central incisors.

**Table 3:** Patient characteristics in each group.

Characteristics	Retreatment Group (*n* = 9)	Control Group (*n* = 12)	*p*-Value
Sex (Female), n (%)	9 (100%)	12 (100%)	N/A ^a^
Age (y)	27.11 ± 8.19	25.08 ± 4.78	0.484
ANB angle (°)	5.79 ± 2.24	4.54 ± 1.82	0.173

Data are presented as mean ± standard deviation or number (percentage). Continuous variables (age, ANB angle) were compared using an independent samples *t*-test. ^a^ Statistical test could not be performed as there was no variation in sex between the groups.

**Table 4:** Comparison of archform and occlusal variables between groups at each stage and between *T*1 and *T2* for the retreatment group.

Variables	Retreatment Group (*n* = 9)				Control Group (*n* = 12)		Comparison Between Groups			
	T0	T1			T0	T1	T0		T1	
	Mean ± SD	Mean ± SD	d	*p* Value	Mean ± SD	Mean ± SD	d	*p* Value	d	*p* Value
Transverse (mm)										
Mx-ICW	34.38 ± 2.30	35.45 ± 0.81	1.07	0.251	34.65 ± 1.85	35.70 ± 1.85	−0.27	0.768	−0.25	0.711
Mx-IPMW	41.71 ± 2.39	43.93 ± 0.90	2.22	0.028 *^‡^	46.62 ± 2.60	44.20 ± 1.41	−4.90	<0.001 ***^‡^	−0.27	0.619
Mx-IMW	46.91 ± 2.31	48.60 ± 1.06	1.69	0.021 *^‡^	51.76 ± 2.47	49.14 ± 1.58	−4.85	<0.001 ***^‡^	−0.54	0.392
Md-ICW	25.81 ± 1.42	27.01 ± 1.17	1.2	0.106 ^†^	26.76 ± 1.72	27.05 ± 0.95	−0.95	0.196 ^†^	−0.04	0.934
Md-IPMW	33.65 ± 1.82	35.07 ± 0.97	1.41	0.062 ^†^	39.02 ± 3.39	36.27 ± 0.88	−5.37	<0.001 ***^‡^	−1.20	0.008 **^‡^
Md-IMW	39.73 ± 2.69	40.30 ± 1.35	0.58	0.516	43.89 ± 2.48	40.82 ± 1.70	−4.17	0.002 **^‡^	−0.52	0.463
Sagittal (mm)										
Mx-AAD	8.87 ± 2.17	7.63 ± 0.55	−1.24	0.084 ^‡^	6.52 ± 2.40	7.92 ± 0.90	2.35	0.032 *^†^	−0.29	0.403
Mx-AD	40.35 ± 3.11	39.50 ± 1.77	−0.86	0.183	45.40 ± 2.83	39.58 ± 1.92	−5.04	0.001 **^‡^	−0.08	0.926
Mx-AP	63.97 ± 2.08	62.04 ± 1.11	−1.92	0.020 *^‡^	75.16 ± 3.72	61.82 ± 3.58	−11.12	0.002 **^‡^	0.23	0.857
Md-AAD	5.92 ± 1.32	5.81 ± 0.85	−0.11	0.764	4.00 ± 1.88	5.39 ± 0.60	1.92	0.003 **^‡^	0.43	0.194 ^†^
Md-AD	37.47 ± 1.82	38.35 ± 2.58	0.88	0.051 ^†^	43.14 ± 2.24	37.47 ± 1.80	−5.67	0.004 **^‡^	0.89	0.365
Md-AP	52.75 ± 1.83	52.37 ± 2.76	−0.38	0.589	64.58 ± 3.26	52.45 ± 2.45	−11.83	0.005 **^‡^	−0.09	0.941
Variable of arch curvature (°)										
Mx-ICA	123.07 ± 9.65	131.74 ± 3.97	8.67	0.011 *^‡^	138.18 ± 13.39	130.46 ± 4.86	−15.11	0.010 *^‡^	1.28	0.528
Md-ICA	128.80 ± 10.57	132.75 ± 3.76	3.95	0.159	145.81 ± 13.67	137.93 ± 6.08	−17.01	0.006 **^‡^	−5.18	0.037 *^‡^
Vertical and Occlusal (mm)										
Curve of Spee	2.44 ± 0.68	0.86 ± 0.39	−1.58	<0.001 ***^‡^	1.89 ± 0.75	0.62 ± 0.43	0.55	0.102 ^†^	0.24	0.211
OJ	5.73 ± 1.64	2.24 ± 0.82	−3.48	0.001 **^‡^	3.91 ± 2.25	2.24 ± 0.86	1.82	0.055 ^‡^	0.00	0.998 ^†^
OB	5.85 ± 1.65	2.35 ± 0.77	−3.5	<0.001 ***^‡^	2.45 ± 1.30	1.84 ± 0.63	3.40	<0.001 ***^‡^	0.51	0.109 ^†^

Mx, maxillary; Md, mandibular; ICW, intercanine width; IPMW, interpromolar width; IMW, intermolar width; AAD, anterior arch depth; AD, arch depth; AP, arch perimeter; ICA, intercanine angle; OJ, overjet; OB, overbite. * *p* < 0.05, ** *p* < 0.01, and *** *p* < 0.001. ^†^ *Hedges’ g* > 0.5; ^‡^ *Hedges’ g* > 0.8.

**Table 5:** Comparison of tooth inclination variables between groups at each stage and between *T*1 and *T*2 for the Retreatment Group.

Variables	Retreatment Group (*n* = 18)				Control Group (*n* = 24)		Comparison Between Groups			
	T0	T1			T0	T1	T0		T1	
	Mean ± SD	Mean ± SD	d	*p* Value	Mean ± SD	Mean ± SD	d	*p* Value	d	*p* Value
MA (°)										
Mx Canine	5.96 ± 6.26	10.91 ± 4.96	4.95	0.010 *^†^	15.14 ± 4.86	7.21 ± 6.01	−9.18	<0.001 ***^‡^	3.70	0.040 *^†^
Mx Premolar	−1.73 ± 4.59	−6.03 ± 5.81	−4.29	0.002 **^‡^	−0.68 ± 4.52	−2.29 ± 4.41	−1.05	0.462	−3.74	0.023 *^†^
Mx First molar	−7.61 ± 5.09	−10.69 ± 8.11	−3.08	0.036 *^†^	−6.38 ± 6.71	−8.27 ± 8.18	−1.23	0.521	−2.43	0.346
Md Canine	17.39 ± 10.96	25.45 ± 6.99	8.06	<0.001 ***^‡^	28.11 ± 8.11	25.64 ± 8.38	−10.71	0.001 **^‡^	−0.19	0.939
Md Premolar	16.03 ± 8.40	14.77 ± 5.09	−1.27	0.412	16.87 ± 4.41	15.70 ± 4.90	−0.84	0.679	−0.94	0.55
Md First molar	14.33 ± 5.78	11.50 ± 3.45	−2.84	0.040 *	14.67 ± 5.70	10.66 ± 8.20	−0.34	0.852	0.83	0.688
BI (°)										
Mx Central Incisor	19.25 ± 7.94	18.06 ± 7.94	−1.19	0.502	25.45 ± 9.82	16.19 ± 6.80	−6.20	0.034 *^†^	1.86	0.418
Mx Canine	3.10 ± 5.03	1.86 ± 5.74	−1.24	0.397	4.89 ± 4.48	0.92 ± 7.15	−1.79	0.232	0.94	0.651
Mx Premolar	−2.42 ± 5.25	0.97 ± 5.07	3.39	0.008 **^†^	−2.53 ± 6.27	−2.01 ± 5.65	0.11	0.951	2.98	0.085 ^†^
Mx First molar	−5.92 ± 5.62	−1.94 ± 5.57	3.98	0.003 **^†^	−2.57 ± 6.11	−4.03 ± 6.86	−3.34	0.077 ^†^	2.10	0.295
Md Central Incisor	28.05 ± 7.76	35.33 ± 5.87	7.28	<0.001 ***^‡^	36.05 ± 5.44	30.85 ± 10.18	−8.00	<0.001 ***^‡^	4.48	0.103 ^†^
Md Canine	4.99 ± 4.79	7.00 ± 3.62	2.01	0.13	7.48 ± 6.60	2.83 ± 4.29	−2.49	0.184	4.17	0.002 **^‡^
Md Premolar	−9.81 ± 8.80	−6.03 ± 5.80	3.78	0.009 **^†^	−7.91 ± 7.53	−2.08 ± 6.97	−1.90	0.462	−3.95	0.058 ^†^
Md First molar	−20.26 ± 6.86	−19.17 ± 5.46	1.09	0.519	−17.58 ± 8.33	−19.00 ± 8.47	−2.68	0.273	−0.17	0.943

Mx, maxillary; Md, mandibular; MA, mesiodistal angulation; and BI, buccolingual inclination. d represents the mean difference between groups (retreatment minus control) for between-group comparisons, and the mean change (*T*2 minus *T*1) for within-group comparisons. * *p* < 0.05, ** *p* < 0.01, and *** *p* < 0.001. ^†^ *Hedges’ g* > 0.5; ^‡^ *Hedges’ g* > 0.8.

## Data Availability

The data presented in this study are available on request from the corresponding author due to privacy.

## Abbreviations

The following abbreviations are used in this manuscript:

AD	Arch depth
AAD	Anterior arch depth
AP	Arch perimeter
BI	Buccolingual inclination
CBCT	Cone-beam computed tomography
FH	Frankfort horizontal
ICA	Intercanine angle
ICW	Interpremolar width
IMW	Interpremolar width
IPMW	Interpremolar width
MA	Mesiodistal angulation
OP	Occlusal plane
TADs	Temporary anchorage devices
